# Exploring the link between innate immune activation and thymic function by measuring sCD14 and TRECs in HIV patients living in Belgium

**DOI:** 10.1371/journal.pone.0185761

**Published:** 2017-10-19

**Authors:** Adrien De Voeght, Henri Martens, Chantal Renard, Dolores Vaira, Mathieu Debruche, Julie Simonet, Vincent Geenen, Michel Moutschen, Gilles Darcis

**Affiliations:** 1 Infectious Diseases Department, University Hospital Center of Liège, University of Liège, Liège, Belgium; 2 Laboratory of Immunology and Infectious Diseases, GIGA-Inflammation, Infection & Immunity, GIGA Research Institute, University of Liège, Liège, Belgium; 3 Department of Biomedical and Preclinical Sciences, GIGA-I 3 Center of Immuno-endocrinology, GIGA Research Institute, University of Liège, Liège, Belgium; 4 AIDS Reference Center, University Hospital Center of Liège, University of Liège, Liège, Belgium; Karolinska Institutet Department of Medicine Solna, SWEDEN

## Abstract

Microbial translocation is now viewed as a central event in the pathogenesis of chronic inflammation during HIV infection. Thymic function failure is another crucial factor involved in HIV disease progression. The goal of this study was to explore the hypothesis of potential links between microbial translocation and thymic function in HIV-1 patients living in Belgium. The extent of microbial translocation was assessed through the measurement of soluble CD14 (sCD14). T-cell receptor excision circles (sjTRECs and dβTRECs) were used as a measure of thymic function. Data were collected from 75 HIV-infected patients. Simple and complex linear regressions were done to analyze the link between these two processes. We found a statistically relevant negative correlation between thymopoiesis (sjTREC) and sCD14 level (p = 0.004). These results suggest a link between thymic function failure, microbial translocation and innate immune activation.

## Introduction

Human Immunodeficiency Virus (HIV) disease progression is led both by viral replication and by immune activation. Through mechanisms unrelated to higher virus burden, immune activation is a major determinant of survival in advanced HIV-1 disease [1].

HIV infection of the gut selectively depletes IL-17-expressing CD4+ T lymphocytes (Th17 cells) present in the gut-associated lymphoid tissue (GALT) [2–4]. Th17 cells produce IL-22, which enhances epithelial regeneration and, as a possible consequence of their loss, impaired mucosal restoration and subsequent increased intestinal permeability and microbial translocation (MT) occur [4–5]. Additionally, exposure to HIV-1 can directly breach the integrity of mucosal epithelial barrier, allowing translocation of bacteria [6].

MT has been viewed for years as a possible mechanism underlying the persistent chronic immune activation associated with HIV infection despite highly active antiretroviral therapy (HAART) [2, 7]. MT is usually evaluated by measuring blood concentration of bacterial lipopolysaccharide (LPS) or 16S rDNA. Nevertheless, measuring LPS concentration is a technically complex process which is difficult to implement in routine care. Some authors have therefore proposed soluble CD14 (sCD14), a marker of monocyte activation, as an indirect marker of M`T since sCD14 is upregulated in response to LPS stimulation [8]. CD14 is a co-receptor for LPS along with toll-like receptor-4 and myeloid differentiation factor-2. It is bound to the membrane by a glycosylphosphatidylinositol anchor. After exposure to bacterial endotoxin, monocytes release sCD14 by a protease-dependent shedding of the membrane form [9] but also by direct secretion of the soluble form [10]. Hepatocytes also produce sCD14 after LPS exposure by both mechanisms [11]. Accordingly, the increase in sCD14 has been described in Gram-negative bacterial sepsis [10] as well as in other conditions associated with MT such as insulin resistance [11], liver inflammation [12], and cardiovascular disease [13]. Several studies have also shown that levels of sCD14 in HIV-infected patients were strongly correlated with endotoxin levels [14–16]. Soluble CD14 can therefore been considered as an indirect biomarker of MT associated with HIV infection [16]. Importantly, from a nested case–control study performed on patients from the SMART trial, it was demonstrated that sCD14 is an independent marker of mortality [8] and has been proposed as a follow-up marker in HIV patients [17].

Otherwise, the effects of HIV infection upon the thymus have been implicated in the pathogenesis of AIDS [18]. HIV infection rapidly induces a substantial suppression of thymocyte proliferation which contributes to the loss of naïve T cells [19]. A direct measure of thymic function is not reasonably practicable. However, in the thymus, T-cell receptor (TCR) chain loci undergo rearrangement of their different gene segments, ultimately leading to the generation of highly diverse CDR3 regions [19]. By-products of these processes, TCR excision circles (TRECs), persist in recent thymic emigrants (RTE). Therefore, TRECs can be used as a surrogate marker for thymic output [20]. Single-joint TRECs (sjTRECS) is the most used technique to measure thymic output. However, sjTRECs are not replicated in the cell cycle and the dilution of this marker may arise due to lymphocyte proliferation [21,22]. The ratio sjTREC/ dβTREC is independent of peripheral proliferation and is therefore another important parameter to evaluate thymic function [23].

To the best of our knowledge, there are but a few works that explore a link between MT and the thymic function. First, LPS induces thymic atrophy in rats [24]. Secondly, a curious medical condition known as idiopathic CD4 lymphocytopenia (ICL) in HIV-negative patients is associated with MT [25]. Importantly, ICL seems to be linked with a decreased thymic function [26].

In this study, we have explored potential links between these two key processes involved in HIV pathogenesis by measuring sCD14 and TRECs in HIV-1 infected patients.

## Results

The study was based on a sample of 75 HIV-1 chronically-infected HAART-treated patients followed at the Liège AIDS Reference Center. The population was composed of 27 Caucasians and 48 black Africans living in Belgium. Blood samples were collected between January 2012 and May 2013. General data are summarized in S1 Table and patients’ characteristics are presented in S2 Table.

### Impact of patients’ characteristics on blood markers

**Fig 1:**
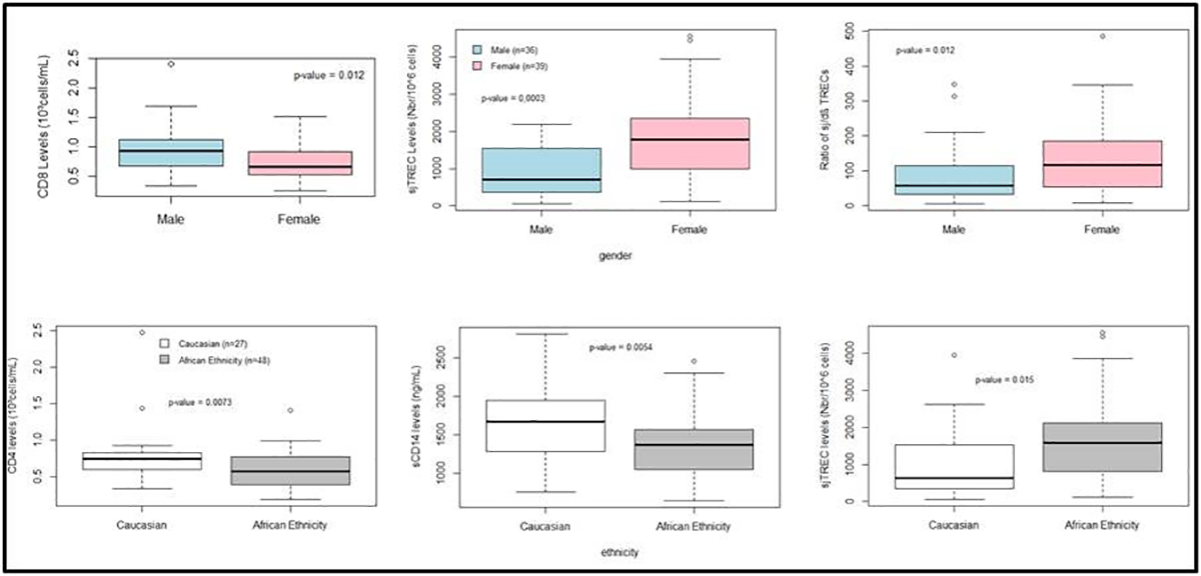
General comparison of data selected for the sex or the ethnicity. A. Impact of the sex on some observed data. Men have higher CD8 (p = 0.012) levels but lower TRECsj (p = 0.0003) and ratio of TREC (p = 0.012) than women. B. Impact of the ethnicity. Caucasians have higher CD4 (p = 0.0073) levels and sCD14 (p = 0.0054) but lower TRECsj (p = 0.015) than people from African Origin.

Gender was associated with different levels of measured values (Fig 1A). Men had higher CD8 levels (p = 0.012) but lower sjTRECs (p = 0.0003) and sj/dβTREC ratio (p = 0.012) than women (Table 1).We further discuss this surprising difference between sexes on TRECs levels in the discussion section.

**Table 1:** Impact of the sex on observed blood markers of 75 HIV+ subjects.

	*Men (N = 36)*	*Women (N = 39)*	*Comparison*
	*Median (IQR)*	*Median (IQR)*	*p-values*
CD4			
Number/μl	370 (540–820)	600 (360–600)	0.090
%	31.0 (26.0–38.0)	30.0 (26.0–38.0)	0.79
CD8			
**Number/μl**	**940 (690–1100)**	**660 (520–920)**	**0.012**
%	41.0 (35.0–47.0)	36.0 (30.5–47.5)	0.17
Ratio CD4/CD8	0.79 (0.61–1.0)	0.93 (0.55–1.1)	0.38
sCD14 (ng/ml)	1392 (1146–1898)	1424 (1102–1618)	0.78
**sjTREC (Nbr/10**^**6**^**cells)**	**706 (371–1532)**	**1773 (991–2356)**	**0.0003**
dβTREC(Nbr/10^6^cells)	11.0 (8.0–17.5)	13.0 (8.0–18.0)	0.38
**Ratio sjTREC/ dβTREC**	**58.5 (33.3–113.8)**	**116.0 (54.5–186.5)**	**0.012**

Ethnicity was also associated with some differences (Fig 1B). Patients of African descent had lower CD4 (p = 0.0073) and sCD14 (p = 0.0054) levels and higher sjTRECs (p = 0.015) compared to Caucasian patients (Table 2). Differences in sCD14 levels between Caucasians and African patients observed in this study are consistent with our previous work [27] showing that Africans patients have lower sCD14 levels.

**Table 2:** Impact of ethnicity on observed blood markers of 75 HIV+ subjects.

	*Caucasian (N = 27)*	*AfricanOrigin (N = 48)*	*Comparison*
*Markers*	*Median (IQR)*	*Median (IQR)*	*p-values*
CD4			
**Number/μl**	**750 (610–820)**	**570 (390–780)**	**0.0073**
%	33.0 (27.5–39.5)	30.0 (24.8–38.0)	0.19
CD8			
Number/μl	850 (610–1100)	740 (530–990)	0.26
%	38.5 (31.5–43.8)	41.5 (32.5–48.3)	0.34
Ratio CD4/CD8	0.88 (0.76–1.1)	0.76 (0.53–1.0)	0.23
**sCD14 (ng/ml)**	**1670 (1286–1945)**	**1375 (1066–1556)**	**0.0054**
**sjTREC (Nbr/10**^**6**^**cells)**	**628 (344–1536)**	**1576 (839–2092)**	**0.015**
dβTREC(Nbr/10^6^cells)	10.0 (7.5–14.5)	15.0 (8.8–24.3)	0.068
Ratio sjTREC/ dβTREC	71.0 (38.0–133.5)	97.5 (42.8–165.2)	0.27

Levels of sCD14 increased in elderly patients (p = 0.049). sjTRECs and sj/dβ TREC ratio decreased with age (p<0.0001 and p<0.0001 respectively). Age-related regression of the thymus is indeed a well-known phenomenon associated with a decline in naïve T cell output [28]. This is thought to contribute to the reduction in T cell diversity seen in older individuals and linked with increased susceptibility to infection, autoimmune disease, and cancer.

Age had no statistically relevant association with other marker levels.

No other significant correlation was found in our data (S1 Fig).

### Relation between sCD14 and TRECs

A simple linear regression was performed between sCD14 and TRECs (Table 3). sjTREC had a significant linear negative correlation with sCD14 (Coef ± SE: -0.11 ± 0.038, p = 0.004). Soluble CD14 decreased when sjTRECs increased.

**Table 3:** Impact of TRECs on sCD14* (N = 75).

*Markers*	*Variables*	*Coef ± SE*	*p-values*
sCD14	Intercept	3.5 ± 0.12	-
	**sjTREC**	**-0.11 ± 0.038**	**0.004**
sCD14	Intercept	3.3 ± 0.060	-
	dβTREC	-0.10 ± 0.053	0.055
sCD14	Intercept	3.2 ± 0.074	-
	Ratio sjTREC/ dβTREC	-0.050 ± 0.038	0.20

* Models of simple linear regression on logarithmic transformed data.

When a multi-variable linear regression was performed, including other patient variables (ethnicity, gender, age), sjTREC still showed a significant linear correlation with sCD14 (Coef ± SE: -0.10 ± 0.046, p = 0.030).

No significant correlation was found between sCD14 and either dβTRECs or sj/dβTREC ratio.

In a complex regression, ethnicity also showed association with sCD14 and sjTRECs. Details are presented in Table 4.

**Table 4:** Impact of TRECs on sCD14 (N = 75), taking into account patients’ characteristics*.

*Markers*	*Variables*	*Coef ± SE*	*p-values*
sCD14	Intercept	3.4 ± 0.20	-
	Age	0.002 ± 0.003	0.48
	**Sex (1 = women)**	**0.072 ± 0.033**	**0.033**
	**Ethnicity (1 = African)**	**-0.093 ± 0.033**	**0.007**
	**sjTREC**	**-0.10 ± 0.046**	**0.030**
sCD14	Intercept	3.1 ± 0.10	-
	**Age**	**0.005 ± 0.002**	**0.037**
	Sex (1 = women)	0.052 ± 0.032	0.11
	**Ethnicity (1 = African)**	**-0.093 ± 0.034**	**0.008**
	dβTREC	-0.083 ± 0.050	0.10
sCD14	Intercept	3.1 ± 0.16	-
	Age	0.004 ± 0.003	0.14
	Sex (1 = women)	0.055 ± 0.034	0.11
	**Ethnicity (1 = African)**	**-0.10 ± 0.034**	**0.003**
	Ratio sjTREC/ dβTREC	-0.023 ± 0.043	0.59

* Models of multiple linear regression on logarithmic transformed data.

## Discussion

Chronic inflammation plays an active role in HIV pathogenesis by impeding the generation of optimal HIV-1-specific immune responses through multiple mechanisms including expansion of T regulatory cells, upregulation of negative regulators on effectors cells and lymphoid organs fibrosis [29]. Chronic inflammation might also lead to HIV persistence by generating new target cells, enabling infection of activated and resting target cells and increasing the proliferation of infected cells [30]. In this regard, the understanding of the mechanisms underlying chronic inflammation and their connections with immune dysfunction is undoubtedly a priority in the HIV field.

MT is a key component of this deleterious chronic immune activation that persists despite suppressive HAART. The extent of MT can be assessed by various laboratory techniques. Most of them such as LPS measurements are complex and difficult to implement in daily routine. As the level of sCD14 is linked to the activation of Toll-Like Receptor 4 (receptor of LPS), it has been considered as a potential marker of MT, with the crucial advantage to be easily measured. Although the link between sCD14 and MT is complex and affected by genetic [31] and ethnicity [27], sCD14 is considered as a good surrogate marker for MT and innate immune activity.

In this study, we looked at the potential relationship between sCD14 and thymic function evaluated by two types of TRECs. Interestingly, and despite the relative low number of tested patients, sjTREC frequency was inversely correlated to sCD14 (p = 0.004). Taking into account patients’ characteristics such as age, sex and ethnicity, the correlation was still statistically relevant (p = 0.03). No significant association between sCD14 and other studied markers of thymic function was demonstrated.

Otherwise, we confirmed the correlation previously observed between ethnicity and the level of sCD14 (p = 0.0054) [27]. Ethnicity was also significantly associated with sjTREC values (p = 0.015).These results suggest that patients of African ethnicity have lower sCD14 levels and higher sjTREC level than Caucasian patients. Interestingly, we observed that thymic function is also lower in males. This surprising observation has been also recently made by Ferrando-Martinez and colleagues and could rely on hormone levels since previous studies showed enhanced thymic function following sex steroid ablation [32, 33]

Soluble CD14 was first described in the host-response to endotoxin (LPS) exposure [9, 10]. Even if sCD14 is considered as an acute-phase protein [34], its biological relevance remains incompletely explained.

Soluble CD14 seems to present two opposite functions regarding to LPS metabolism. First, it decreases activity of LPS by contributing to remove LPS from the circulation [35]. Soluble CD14 further reduces the LPS-induced monocyte activation by competing with CD14 receptor for binding LPS [36]. Nevertheless, sCD14 seems to induce activation of cells that do not substantially express CD14 when they are exposed to LPS [37, 38]. Low concentration of sCD14 is associated with a pro-inflammatory state due to the release of inflammatory cytokines as well as endothelial, epithelial and smooth muscle activation [37–39]. Taken together, these findings suggest that the moderate to high concentrations of sCD14 that are found in blood may help to prevent LPS-induced systemic inflammation, whereas lower concentrations of this protein, which presumably occur in extravascular fluids, may promote beneficial inflammation at local sites of infection [40].

Thymic involution and impairment of thymopoiesis are well-known consequences of HIV infection [41]. According to previous reports, HIV-infection is associated with a substantial reduction of intrathymic proliferation [18, 19]. Moreover, Sauce *et al*. demonstrated that HIV-associated CD4+ T cells depletion has also a more upstream cause [42]. They showed that HIV disrupts central lymphopoiesis independently of viral replication. They further observed a negative correlation between sCD14 and the CD34+ hematopoietic progenitor cells compartment (primary source of all lymphocytes), suggesting that chronic immune activation and MT participate in disrupting central lymphopoeisis [42].

However, to the best of our knowledge, the association between MT and thymic function has been poorly studied. Only two studies have explored the hypothesis of a relationship between MT and thymic function. Owens and Berg showed an incidence of bacterial translocation of 50% in athymic mice [43].The incidence of bacterial translocation was reduced to 7.8% following thymus graft, highlighting the role of T-cell-dependent immunity in preventing bacteria from translocating from the gastrointestinal tract [43]. Otherwise, Wang et al. demonstrated that LPS injections to induce a sepsis lead to apoptosis of thymocytes in mice [44]. All these observations therefore suggest some complex interaction between MT and thymic function.

Our hypothesis of the relationship between MT and thymic function is the following: HIV infection induces severe damages in peripheral and central immune system. This impairment of adaptive immunity by loss of functional CD4+ T cells increases MT. In return, MT may activate innate immunity (both reflected by an increase in sCD14 levels), participating in thymopoiesis disruption despite suppressive HAART.

This relation could have direct clinical impacts. Indeed, immune activation and the level of CD4+ T lymphocytes are two major concerns for the follow-up of HIV patients. The measure of sCD14 level seems to link these two parameters. So its implementation in routine analyses could help clinicians to understand/predict/prevent the emergence of various disorders associated with HIV-1 infection.

In conclusion, we demonstrated a statistically-relevant negative correlation between sCD14 and thymic function in HIV-1 patients living in Belgium. These results highlight that MT and disrupted thymic function participate to a complex vicious circle resulting in persistent immune activation and immune dysfunction despite suppressive HAART.

## Materials and methods

The Ethical Committee of the University Hospital Center of Liège approved the study protocol. Written consent for participation was obtained in accordance with institutional review board standards according to the Declaration of Helsinki.

### Biomarker measurements

#### DNA extraction

10 ml of blood were withdrawn on EDTA tube. Following centrifugation, 250 μL of buffy coat were used to perform the DNA extraction.

Extraction has been performed using the “automated extraction of DNA from blood or buffy coat samples kit” (The Maxwell® Blood DNA Purification Kitfrom Promega).

DNA concentration was estimated by measuring the absorbance at 260nm.

#### Analysis of thymus function (sjTREC and dβTRECs)

Measurement of TRECs was based on our previously published methodology [45]. All primers were purchased from Eurogentec (Seraing, Belgium).Two plasmids were used to generate standard curves for real-time quantitative PCR-based assay, each containing two inserted amplicons (CD3γ with either sjTREC or Dβ1–Jβ1.4) amplified in the same run as experimental samples.

Parallel quantification of each TREC together with the CD3γ amplicon as single-copy gene was performed for each sample providing an absolute number of TRECs per 10^6^ cells. Multiplex PCR amplification was performed for sjTRECs, together with the CD3γ chain for 22 cycles using the ‘out’ 3’/5’ primer pairs. Following the first round of amplifications, PCR products were diluted prior to online real-time amplification using LightCycler 480 instrument (Roche Diagnostics, Basel, Switzerland). For each PCR product, the TREC and CD3γ second-round PCR quantifications were performed in separate well on the same plate and quantified on the same first-round serially diluted standard curve. The results are expressed as an absolute number of TRECs per 10^6^ cells.

#### sCD14 analysis

sCD14 was measured by using enzyme-linked immunosorbent assay with the manufacturers’ protocol (R&D Systems, Minneapolis, Minnesota, USA) on 100μL of patient’s serum (serum diluted to 0,5%).

### Statistical analyses

Results were summarized as follows: quantitative data were expressed in terms of mean, standard deviation (SD), median, interquartile range (IQR) and extreme values while qualitative data were expressed in terms of frequency table.

The Spearman correlation coefficient was calculated to measure the association between two quantitative variables. A matrix of correlation was calculated using the Spearman method.

The impact of variables such as age, sex and ethnicity (African Origins or Caucasians) on blood markers were calculated:

▪ The impact of age on blood markers was calculated by linear regression (after the data set has undergone a logarithmic transformation).
▪ A non-parametric Kruskal-Wallis test [46] was used to analyze sex and ethnicity.

The relationship between sCD14 and TRECs was computed using a linear regression (after the data set has undergone a logarithmic transformation) with (multiple linear regression) and without (simple linear regression) co-variables characterizing the patient (age, sex and ethnicity).

Calculations were computed on the maximum of available data. Not a single missing value was replaced. Results were considered significant at the 5% critical level (P < 0.05). The analyses were carried out using R (version 3.0.3) and RStudio (version 3.2.0) statistical packages [47].

## Supporting information

S1 Fig Overview of the data, correlation matrix (Spearman method). (DOCX)

S1 Table Description of patients and the observed markers (N = 75). (DOCX)

S2 Table Presentation of patients characteristics. Characteristics (age, CD4+T cell count, CD4+ nadir, antiviral regimens, duration of therapy, plasma HIV-1 RNA level, year of diagnosis) of patients from the Liege AIDS reference center (N = 75) are presented. “NT” indicates “Not Treated”. “NA” indicates “Not Applicable”.
(DOCX)
