# Supplementary figures and images for: Exploring the link between innate immune activation and thymic function by measuring sCD14 and TRECs in HIV patients living in Belgium
Source: PLoS One. 2017 Oct 19;12(10):e0185761. doi: 10.1371/journal.pone.0185761 (PMC5648129; doi:10.1371/journal.pone.0185761)

**Supporting Information**

**S1 Fig. Overview of the data, correlation matrix (Spearman Method)**


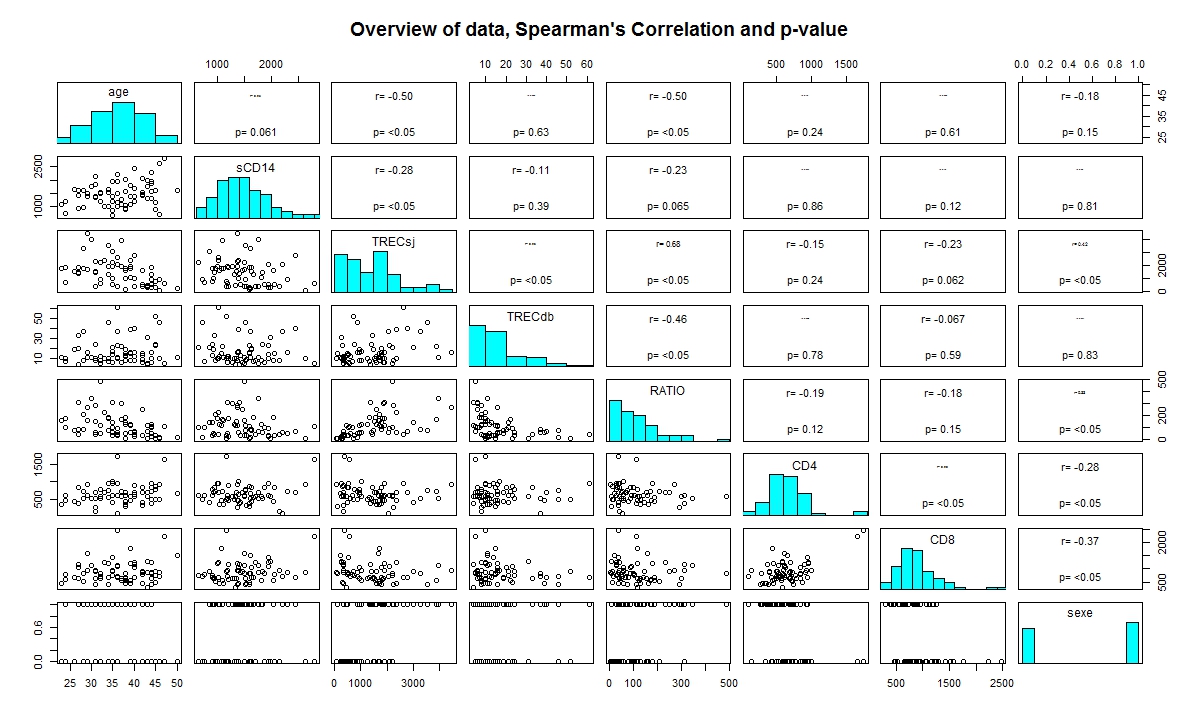

Supplement: S1 Fig — (DOCX) [file pone.0185761.s001.docx]
