# Supplementary material for: Exploring the link between innate immune activation and thymic function by measuring sCD14 and TRECs in HIV patients living in Belgium
Source: PLoS One. 2017 Oct 19;12(10):e0185761. doi: 10.1371/journal.pone.0185761 (PMC5648129; doi:10.1371/journal.pone.0185761)
Supplement: S1 Table — (DOCX) [file pone.0185761.s002.docx]

**Supporting Information**

**S1 Table . Description of patients and the observed markers (N=75)**

|  | *N (%)* | *Mean ± SD* | *Median (IQR)* | *Extremes* |
| --- | --- | --- | --- | --- |
| Age (years) | 75 | 37 ± 7 | 37 (32 – 43) | 23 - 50 |
|  |  |  |  |  |
| Sex |  |  |  |  |
| Men | 36 (48) |  |  |  |
| Women | 39 (52) |  |  |  |
|  |  |  |  |  |
| Ethnicity |  |  |  |  |
| Caucasian | 27 (36) |  |  |  |
| AfricanOrigin | 48 (64) |  |  |  |
|  |  |  |  |  |
| CD4 |  |  |  |  |
| Number/µl | 73 | 660 ± 320 | 620 (440 – 790) | 200 – 2500 |
| % | 74 | 31.8 ± 8.9 | 30.5 (26.0 – 38.0) | 10.0 – 52.0 |
| CD8 |  |  |  |  |
| Number/µl | 73 | 840 ± 380 | 780 (550 – 1100) | 250 – 2400 |
| % | 74 | 39.9 ± 10.0 | 39.5 (31.5 – 47.0) | 21.0 – 59.0 |
| Ratio CD4/CD8 | 74 | 0.89 ± 0.44 | 0.86 (0.57 – 1.1) | 0.22 – 2.4 |
|  |  |  |  |  |
| sCD14 (ng/ml) | 75 | 1471 ± 453 | 1409 (1108 – 1747) | 655 – 2803 |
|  |  |  |  |  |
| sjTREC (Nbr/10^6^cells) | 75 | 1402 ± 1048 | 1316 (534 – 1874) | 68 – 4556 |
| dβTREC (Nbr/10^6^cells) | 75 | 16.6 ± 12.4 | 12.0 (8.0 – 21.0) | 3.0 – 61.0 |
| Ratio sjTREC/ dβTREC | 75 | 113.2 ± 97.4 | 84.0 (41.5 – 155.5) | 7.0 – 485.0 |
