# Supplementary material for: Exploring the link between innate immune activation and thymic function by measuring sCD14 and TRECs in HIV patients living in Belgium
Source: PLoS One. 2017 Oct 19;12(10):e0185761. doi: 10.1371/journal.pone.0185761 (PMC5648129; doi:10.1371/journal.pone.0185761)
Supplement: S2 Table — Characteristics (age, CD4+T cell count, CD4+ nadir, antiviral regimens, duration of therapy, plasma HIV-1 RNA level, year of diagnosis) of patients from the Liege AIDS reference center (N = 75) are presented. “NT” indicates “Not Treated”. “NA” indicates “Not Applicable”. (DOCX) [file pone.0185761.s003.docx]

**S2 Table. Presentation of patient characteristics.**

Characteristics (age, CD4+T cell count, CD4+ nadir, antiviral regimens, duration of therapy, plasma HIV-1 RNA level, year of diagnosis) of patients from the Liege AIDS reference center (N=75) are presented. “NT” indicates “Not Treated”. “NA” indicates “Not Applicable”.

| **Patients** | **Age** | **Year of blood sampling** | **Last ART** | **Year of diagnosis** | **CD4+ T cell count (cells/mm3)** | **Viral load** | **CD4+ T cell Nadir** | **Year of 1st treatment** |
| --- | --- | --- | --- | --- | --- | --- | --- | --- |
| P1 | 34 | 2012 | FTC NVP TDF | 1996 | 885 | 112 | 308 | 1998 |
| P2 | 39 | 2012 | FTC NVP TDF | 2009 | 654 | <50 | 157 | 2009 |
| P3 | 42 | 2012 | ABC 3TC NVP RAL | 2007 | 329 | 3020 | 180 | 2007 |
| P4 | 40 | 2012 | ATV/r FTC TDF | 2004 | 753 | <512 | 506 | 2009 |
| P5 | 33 | 2012 | NT | 2009 | 854 | 310 | 462 | NA |
| P6 | 32 | 2013 | ABC FPV/r 3TC TDF | 2008 | 604 | <50 | 470 | 2010 |
| P7 | 29 | 2012 | FTC NVP TDF | 2010 | 794 | 333 | 321 | 2011 |
| P8 | 41 | 2012 | ATV/r FTC TDF | 1994 | 202 | <50 | 175 | 1994 |
| P9 | 44 | 2012 | ABC ATV/r FTC TDF | 1991 | 435 | 66 | 110 | 1997 |
| P10 | 46 | 2013 | NT | 2000 | 545 | 9840 | 400 | 2000 |
| P11 | 44 | 2012 | ABC ATV/r 3TC | 2006 | 1436 | <50 | 280 | 2006 |
| P12 | 35 | 2012 | ATV/r FTC TDF | 1995 | 908 | 19000 | 337 | 1997 |
| P13 | 34 | 2012 | NT | 2008 | 877 | 25400 | 550 | NA |
| P14 | 50 | 2012 | ABC DRV/r ETR TDF | 2005 | 626 | 69 | 20 | 2005 |
| P15 | 40 | 2012 | ATV/r FTC TDF | 2008 | 762 | 873 | 526 | 2012 |
| P16 | 31 | 2012 | NT | 2011 | 492 | 340000 | 508 | NA |
| P17 | 37 | 2013 | ABC FPV/r 3TC | 2007 | 738 | 124 | 300 | 2008 |
| P18 | 47 | 2012 | ABC ATV/r 3TC | 1998 | 2474 | 6400 | 319 | 1998 |
| P19 | 36 | 2012 | ABC 3TC NVP | 2008 | 732 | <50 | 150 | 2008 |
| P20 | 28 | 2012 | NT | 2010 | 471 | 964 | 419 | 2010 |
| P21 | 37 | 2012 | FTC RAL TDF | 1995 | 688 | <50 | 18 | 1996 |
| P22 | 43 | 2012 | ABC ATV/r 3TC | 1995 | 790 | <50 | 156 | 1995 |
| P23 | 45 | 2012 | 3TC NVP AZT | 2004 | 1122 | <50 | 230 | 2004 |
| P24 | 30 | 2012 | FTC NVP TDF | 2006 | 619 | <50 | 240 | 2006 |
| P25 | 43 | 2012 | ATV/r FTC TDF | 2009 | 465 | <50 | 200 | 2009 |
| P26 | 43 | 2012 | EFV FTC TDF | 2002 | 568 | 164 | 83 | 2002 |
| P27 | 45 | 2012 | FTC NVP TDF | 2000 | 827 | 210 | 564 | 2000 |
| P28 | 38 | 2012 | 3TC NVP AZT | 2004 | 756 | <50 | 320 | 2004 |
| P29 | 39 | 2012 | FTC NVP TDF | 2010 | 434 | <50 | 250 | 2010 |
| P30 | 42 | 2012 | ABC 3TC LPV/r | 2008 | 928 | <50 | 320 | 2009 |
| P31 | 45 | 2012 | EFV FTC TDF | 2003 | 533 | <50 | 0 | 2003 |
| P32 | 32 | 2012 | 3TC NVP AZT | 2000 | 436 | 55 | 88 | 2000 |
| P33 | 28 | 2013 | ATV/r FTC TDF | 2008 | 856 | 593 | 440 | 2009 |
| P34 | 44 | 2012 | ABC ATV/r | 1995 | 573 | 128 | 95 | 1997 |
| P35 | 42 | 2012 | ABC LPV/r TDF | 1993 | 339 | 7860 | 52 | 1998 |
| P36 | 35 | 2012 | NT | 2008 | 578 | 3070 | 578 | NA |
| P37 | 27 | 2012 | ABC 3TC NVP | 2006 | 802 | 302 | 240 | 2007 |
| P38 | 42 | 2012 | FTC LPV/r TDF | 2003 | 896 | <50 | 140 | 2005 |
| P39 | 35 | 2012 | FTC FPV/r TDF | 2003 | 877 | <50 | 25 | 2003 |
| P40 | 34 | 2012 | NT | 2011 | 593 | 1000 | 488 | NA |
| P41 | 23 | 2012 | DRV/r FTC TDF | 2009 | 409 | <50 | 264 | 2011 |
| P42 | 38 | 2012 | FTC LPV/r TDF | 2006 | 593 | <50 | 462 | 2007 |
| P43 | 45 | 2012 | ABC 3TC AZT | 2002 | 990 | <50 | 297 | 2002 |
| P44 | 28 | 2012 | EFV FTC TDF | 2010 | 409 | <50 | 284 | 2010 |
| P45 | 38 | 2012 | ATV/r FTC TDF | 1994 | 218 | <50 | 140 | 1998 |
| P46 | 40 | 2012 | 3TC TDF AZT | 1991 | 772 | <50 | 227 | 2007 |
| P47 | 36 | 2012 | FTC LPV/r TDF | 2011 | 304 | 112 | 98 | 2011 |
| P48 | 39 | 2012 | NT | 2001 | 399 | 886 | 340 | 2002 |
| P49 | 36 | 2012 | FTC NVP TDF | 2003 | 790 | <50 | 290 | 2007 |
| P50 | 46 | 2013 | ATV/r RAL TDF | 1995 | 784 | <50 | 26 | 1996 |
| P51 | 45 | 2012 | ABC ATV/r 3TC | 2004 | 482 | <50 | 144 | 2004 |
| P52 | 27 | 2013 | DRV/r FTC TDF | 2010 | 855 | <50 | 411 | 2011 |
| P53 | 40 | 2012 | 3TC SQV/r AZT | 2004 | 640 | 50 | 127 | 2004 |
| P54 | 39 | 2013 | FTC NVP TDF | 2011 | 369 | 112 | 267 | 2011 |
| P55 | 32 | 2012 | ABC 3TC NVP | 2002 | 814 | 190 | 640 | 2002 |
| P56 | 39 | 2012 | ABC ATV/r 3TC | 2007 | 647 | <50 | 260 | 2009 |
| P57 | 35 | 2012 | ABC ATV/r 3TC | 2003 | 767 | <50 | 200 | 2003 |
| P58 | 35 | 2012 | ATV/r FTC TDF | 2005 | 347 | 37700 | 336 | 2011 |
| P59 | 36 | 2012 | EFV FTC TDF | 2004 | 800 | <50 | 75 | 2004 |
| P60 | 31 | 2012 | FTC NVP TDF | 2009 | 348 | <50 | 140 | 2009 |
| P61 | 29 | 2013 | NT | 2011 | 1408 | 829 | 932 | NA |
| P62 | 35 | 2013 | NT | 2005 | 766 | <50 | 480 | NA |
| P63 | 32 | 2013 | FTC RPV TDF | 2006 | 330 | 104 | 260 | 2010 |
| P64 | 44 | 2012 | FTC FPV/r TDF | 2002 | 602 | <50 | 204 | 2002 |
| P65 | 24 | 2012 | EFV FTC TDF | 2009 | 543 | <50 | 200 | 2010 |
| P66 | 44 | 2012 | ABC 3TC AZT | 1990 | 592 | <50 | 130 | 1997 |
| P67 | 44 | 2012 | 3TC LPV/r | 2001 | 848 | 56 | 12 | 2001 |
| P68 | 24 | 2012 | FTC LPV/r TDF | 2011 | 448 | <50 | 288 | 2011 |
| P69 | 31 | 2013 | 3TC LPV/r RAL | 2005 | 232 | <50 | 20 | 2005 |
| P70 | 36 | 2012 | ATV/r FTC TDF | 2007 | 998 | <50 | 390 | 2009 |
| P71 | 31 | 2012 | 3TC NVP AZT | 2003 | 626 | 1370 | 245 | 2003 |
| P72 | 46 | 2012 | EFV FTC TDF | 2011 | 437 | <50 | 374 | 2012 |
| P73 | 38 | 2013 | NT | 2011 | 342 | <50 | 342 | NA |
| P74 | 26 | 2012 | EFV FTC TDF | 2009 | 718 | <50 | 340 | 2011 |
| P75 | 38 | 2012 | ABC 3TC LPV/r | 2007 | 333 | <50 | 0 | 2009 |
